# Heterologous Expression, Engineering and Characterization of a Novel Laccase of *Agrocybe pediades* with Promising Properties as Biocatalyst

**DOI:** 10.3390/jof7050359

**Published:** 2021-05-04

**Authors:** Pablo Aza, Gonzalo Molpeceres, Francisco Javier Ruiz-Dueñas, Susana Camarero

**Affiliations:** Centro de Investigaciones Biológicas Margarita Salas, CSIC. Ramiro de Maeztu 9, 28040 Madrid, Spain; pabloaza@cib.csic.es (P.A.); gonzalo.molpeceres@cib.csic.es (G.M.); fjruiz@cib.csic.es (F.J.R.-D.)

**Keywords:** laccase, Agaricales, heterologous expression, *S. cerevisiae*, enzyme directed evolution, *N*-glycosylation, biocatalysis

## Abstract

Agaricomycetes fungi responsible for decay of wood and other lignocellulosic substrates constitute a valuable source of lignin-degrading enzymes. Among these enzymes, laccases (multi-copper oxidases) present remarkable biotechnological potential as environmentally friendly biocatalysts able to oxidize a wide range of aromatic compounds using oxygen as the only requirement. Laccases from saprotrophic Agaricales species have been much less studied than laccases from Polyporales, despite the fact that the former fungi are excellent sources of laccases. Here, the gene of a novel laccase of *Agrocybe pediades*, that is secreted by the fungus during lignocellulose degradation, was synthesised de novo and expressed in *Saccharomyces cerevisiae* using an improved signal peptide previously obtained and enzyme directed evolution. The characterization of the new laccase variants provided new insights on the contribution of different amino acid residues to modulate laccase production, catalytic activity or optimal pH. The selected double-mutated variant also showed interesting properties as a biocatalyst, such as the ability to oxidise a wide range of substrates, including high-redox potential mediators and recalcitrant organic dyes, improved activity at neutral pH and high tolerance to inhibitors. Finally, we demonstrate the existence of three *N*-glycosylation sites in the laccase and their distinct effect on the secretion or catalytic activity of the enzyme.

## 1. Introduction

Laccases (EC 1.10.3.2) are multicopper oxidases (MCOs) widely distributed in nature (fungi, bacteria and plants) that catalyse the oxidation of a large variety of organic substrates (substituted phenols, aromatic amines, benzenethiols, heterocycles, etc.) coupled to the reduction of O_2_ to water. They hold four catalytic copper ions in their active site, one copper type 1 (T1), one type 2 (T2) and two T3 coppers. The reducing substrate is oxidized in the T1 site and four electrons are transferred to the T2/T3 trinuclear cluster (TNC), where one molecule of oxygen is reduced to two molecules of water. The oxidation of the substrate at the T1 site and the electron transfer from the T1 site to the TNC is assisted by highly conserved residues coordinating the catalytic coppers [1]. The T1 site is coordinated by two His and one Cys residues, while a total of eight His residues coordinate the TNC coppers. A fourth Met ligand typically binds axially the T1 copper in plant and bacterial laccases, resulting in a tetrahedral geometry, while a non-coordinating Phe or Leu occupies this position in most fungal laccases, resulting in a T1 site with trigonal geometry. The redox potential of the T1 site classifies laccases as low-redox potential (E^°^ < 500 mV), in bacteria and plants, and medium (E^°^ = 500–710 mV) or high-redox potential (E^°^ = 720–800 mV) in fungi [2,3]. Pieces of evidence support the importance of the geometry of the T1 site in tuning the redox potential of laccases [4,5] although this is not the only determinant [6,7,8].

The high redox potential of certain laccases secreted by white-rot fungi like PM1 basidiomycete, or different species of *Trametes* or *Pycnoporus* [9,10,11,12,13], expand their oxidation capabilities to a wider substrate range than their medium- and low-redox potential counterparts [6]. For instance, oxidation of some high-redox potential mediator compounds such as 4-hydroxybenzotriazole (HBT), violuric acid or p-coumaric acid is restricted to high-redox potential laccases (HRPLs) [14,15]. Once oxidized by the enzyme, the redox mediators expand the laccase substrate portfolio by acting as diffusible electron shuttles between the enzyme and the oxidizable compound in the so-called laccase-mediator systems. Therefore, HRPLs are particularly relevant in biotechnology, with applications in organic chemistry, pulp & paper, food and textile industries, bioremediation, and in biosensors and biofuel cells [16,17]. The possibility of adjusting the intrinsic enzyme properties to the industrial requirements through protein engineering promotes the biotechnological potential of these laccases by developing tailor-made biocatalysts for specific applications [18,19,20,21].

Typically, the majority of well-characterised laccases belong to the order Polyporales. This is because Basidiomycota (Agaricomycetes) laccases have been thoroughly studied due to their participation in lignin biodegradation during wood decay, a process that is mainly carried out by white-rot Polyporales species [22]. However, the order Agaricales contains the largest number of known saprotrophic fungal species that have diversified during evolution to colonise a variety of lignocellulosic substrates. Their unparalleled diversity of lifestyles (comprising white-rot, brown-rot, leaf-litter, grass-litter and decayed wood decomposers) have been recently correlated with changes in their enzymatic toolkits of lignocellulolytic oxidoreductases [23]. The high evolutionary rates of class-II peroxidase, laccase, glucose–methanol–choline oxidase, unspecific peroxygenase and lytic polysaccharide monooxygenase gene families, paralleling the ecological diversification in Agaricales, support the relevance of oxidative enzymatic machinery in the evolution of saprotrophic lifestyles in this order. In particular, Agaricales species growing on forest litter, decayed wood and grass litter constitute remarkable sources of laccases, both in number and in the diversity of laccase-like types. One of these 52 Agaricomycetes genomes analysed in that study was *Agrocybe pediades*, a representative Agaricales species growing on pastures and meadows (grass litter lifestyle). The fungus possesses a significant set of MCO genes, most of which are laccases [23].

The objective of this work is to characterise in detail a novel laccase from *A. pediades* specifically selected from the genome of this fungus because it was secreted under ligninolytic conditions during solid-state fermentation on wheat straw. This makes the enzyme an interesting subject of study to evaluate if it might show promising properties as a biocatalyst like other laccases secreted by white-rot Polyporales. The laccase gene was synthesized de novo and expressed in *S. cerevisiae*, and the enzyme was subjected to directed evolution to improve the laccase activity detected in the culture broth. Enzyme directed evolution is a powerful protein engineering approach that mimics the main processes of natural evolution (mutagenesis and selection of the fittest variants) to obtain improved enzymes for specific purposes [24]. The high homologous recombination frequency of *S. cerevisiae* provides important advantages for enzyme directed evolution. Moreover, the yeast is a preferred host for expression and directed evolution of fungal enzymes due to its capability to perform the post-translational modifications required to secret active eukaryotic enzymes [25,26,27]. Glycosylation is one of the major requirements of fungal laccases. Although glycosylation is supposed to play a crucial biological role in enzyme protection against proteolysis and in protein folding [28,29,30], some of its effects are not yet fully understood and contradictory results can be found in the literature. This prompted us to study the influence of the linked sugars on the secretion, activity and stability of the recombinant *A. pediades* laccase. 

## 2. Materials and Methods

### 2.1. Reagents and Strains

Yeast Transformation Kit, 2,6-dimethoxyphenol (DMP), *N*,*N*-dimethyl-1,4-phenylenediamine (DMPD), 5-Hydroxyimino-2,4,6(1H,3H,5H)-pyrimidinetrione (violuric acid), Evans Blue (EB), Reactive Black 5 (RB5), aniline, p-phenylenediamine (PPD) and HBT were purchased from Sigma-Aldrich (St. Louis, MI, USA). High Pure Plasmid Isolation Kit and 2,2′azinobis (3 ethylbenzothiazoline-6 sulphonic acid) (ABTS) were obtained from ROCHE (Basel, Switzerland). Detergents: polyoxyethylene (10) tridecyl (PET), TWEEN 20 and CHAPS were purchased from Sigma-Aldrich (St. Louis, MI, USA). Phusion High-Fidelity DNA polymerase and Restriction enzymes were purchased from New England Biolabs (Ipswich, MA, USA). QIAquick gel extraction kit from Qiagen (Hilden, Germany). ZymoprepTM Yeast Plasmid Miniprep II was purchased from Zymo Research (Irvine, CA, USA). *S. cerevisiae* BJ5465 strain was purchased from LGC Promochem (Teddington, UK) and Mutazyme II DNA polymerase was from Agilent (Santa Clara, CA, USA). *Agrocybe pediades* AH40210 was obtained from the University of Alcalá Herbarium Culture Collection, Alcalá de Henares, Spain.

### 2.2. Culture and Media

Glucose-ammonium medium [31], Minimal medium (MM), EB expression medium [32] and SEM expression medium (without including ethanol) [33] were prepared as already described. For laccase expression EB and SEM were supplemented with 4 mM and 2 mM CuSO_4_, respectively. 

### 2.3. Agrocybe pediades Secretome

The secretome of *A. pediades* AH40210 was collected from cultures in glucose-ammonium medium and on wheat straw as follows. The fungus was grown in 250 mL containing 50 mL of glucose-ammonium medium or 4 g of chopped wheat (*Triticum aestivum*) straw (particle size ~5–20 mm long × 1–3 mm wide) soaked with 10 mL of distilled water. Inoculum for both culture media consisted of 4 mL of homogenized actively growing mycelium from glucose-ammonium cultures (at 180 rpm and 28 °C) washed and resuspended in sterile distilled water. Both liquid and solid-state fermentation cultures were grown at 28 °C under static conditions in the dark. Samples (entire flasks in triplicate) were collected after 6, 14 and 43 days of incubation. Samples from fungal cultures on lignocellulose were treated with 80 mL distilled water at 180 rpm and 24 °C for 100 min. These and the samples from fungal cultures grown on glucose-ammonium medium were filtered under vacuum and the filtrates were used for proteomic analyses. 

Total extracellular proteins in the above filtrates were freeze-dried, resuspended in 20 mM sodium tartrate (pH 5), and the impurities were removed by a short SDS-PAGE (10% polyacrylamide) stained with Coomassie. The protein bands were cut, destained using 50 mM ammonium bicarbonate in 50% acetonitrile and subjected to tryptic digestion [34]. Tryptic peptides were analysed in an LTQ-Orbitrap Velos mass spectrometer coupled to an Easy-nLC 1000 HPLC system (Thermo Scientific, Waltham, MA, USA). Peptides were first loaded into a precolumn Acclaim PepMap 100 (Thermo Scientific, Waltham, MA, USA), and then eluted onto an Acclaim PepMap C18 colum (25 cm long, 75 µm inner diameter and 3 µm particle size) (Thermo Scientific, Waltham, MA, USA) using a 120 min gradient set as follows: 0–35% solvent B for 90 min, 35–45% solvent B for 10 min, 45–95% solvent B for 5 min, 95% solvent B for 10 min, 95–100% solvent B for 1 min and 100% solvent B for 4 min, at a flow rate of 250 nL/min (solvent A: 0.1% formic acid in 2% acetonitrile; solvent B: 0.1% formic acid in pure acetonitrile). Mass spectrometry (MS) analysis was performed in the Orbitrap at 30,000 (at *m/z* 400) resolution using a 200–1600 *m/z* mass range. After the survey scan, the 15 most intense precursor ions were selected for collision-induced dissociation fragmentation in the ionic trap. Fragmentation was performed with a normalized collision energy of 35%. Charge state screening was enabled to reject unassigned and singly charged protonated ions. A dynamic exclusion time of 45 s was used to discriminate against previously selected ions.

The MS data were analysed with Proteome Discoverer (version1.4.1.14) (Thermo Scientific, Waltham, MA, USA) using standardized workflows. Acquired spectra were searched against the catalog of predicted proteins from the *A. pediades* AH40210 genome, available at the JGI fungal genome portal MycoCosm (https://mycocosm.jgi.doe.gov/Agrped1 accessed on 3 May 2021), using the SEQUEST search engine. Precursor and fragment mass tolerance were set to 10 ppm and 0.5 Da, respectively, allowing a maximum of two missed cleavages, carbamidomethylation of cysteines as a fixed modification, and methionine oxidation as a variable modification. Identified peptides were validated using a Percolator algorithm [35] with a q-value threshold of 0.01.

### 2.4. Predictions and Modelling

NetNGlyc 1.0 Server at http://www.cbs.dtu.dk/services/NetNGlyc/ accessed on 3 May 2021 was used for prediction of *N*-glycosylation sites. The 3D molecular structure model of ApL was built with *Trametes trogii* laccase (PDB 2HRG) as template using the Swiss-model server [36]. Analysis of mutations and representation of the 3D protein structures were performed with PyMol. Graphical representation of amino acid frequencies was done with WebLogo [37] and the server ESBRI [38] was used for the evaluation of salt bridges. 

### 2.5. Enzyme Engineering in S. cerevisiae

The coding sequence (CDS) of *A. pediades* laccase (ID 823363, JGI) fused to α_9H2_ signal peptide and cloned in the episomic pJRoC30 vector was obtained in a previous work [39].

Error prone PCR (epPCR) was carried out with Mutazyme II DNA polymerase following seller recommendations and using the ExtFw sense primer and ExtRv antisense primer (Table S1). PCR products were purified using QIAquick gel extraction kit and mutated genes were co-cloned with linearized pJRoC30 vector (BamHI/NotI) in *S. cerevisiae* by IVOE [40].

Site-directed mutagenesis were carried out using customised mutagenic primers (Table S1) to introduce single point mutations. For each mutated site, two fragments were obtained: one with the ExtFw sense and specific mutation antisense primers and the second with the specific mutation sense and ExtRv antisense primers (Table S1). Products were co-cloned in *S. cerevisiae* as mentioned above.

Saturation Mutagenesis (SM) and Combinatorial Saturation Mutagenesis (CSM): two complementary degenerated mutagenic primers were designed for obtaining CSM libraries over 453rd and 454th or 459th and 460th positions, and SM over the fourth axial ligand position of the DM variant (Table S1). PCR and co-cloning methodology were carried out as described above. GLUE-IT was used to determine the number of clones to be screened in order to cover all possible amino acid combinations (coverage at 95% of confidence) [41].

### 2.6. Laccase Expression in Yeast Microfermentations and High-Throughput Screening of Mutant Libraries

Individual colonies from the mutagenic library were picked and transferred to 50 µL of MM in sterile 96-well plates. H1 position was not inoculated (negative control) and column 6 was inoculated with the parent type for comparison. The plates were incubated at 28 °C, 70% humidity, 200 rpm in a humidity shaker (Minitron-INFORS). After 24 h, we added 160 µL of SEM and plates were incubated for another 48 h. Then, plates were centrifugated at 1000× *g*, 4 °C, 10 min and 20 µL aliquots of supernatants were transferred to replica plates. Addition of 180 µL 3 mM ABTS in Citrate-phosphate (CP) buffer at pH 3 or acetate phosphate buffer pH 6 started the enzymatic reactions that were monitored in a plate reader SpectraMax M2 (Molecular Devices, Sunnyvale, CA, USA), in kinetic mode at 25 °C by the increment in absorption at 418 nm (εABTS = 36,000 M^−1^ cm^−1^). The activities of the clones were normalized to the activity of the parent type in each plate. One activity unit (U) is defined as the amount of enzyme needed to transform 1 μmol substrate/minute at room temperature.

First re-screening. Aliquots (5 µL) from selected clone cultures were inoculated in a new sterile 96-well plate together with 50 µL MM in columns 2 and 7. Well D7 was inoculated with the parent type. Columns 1 and 12 and rows A and H were not used. Plates were incubated at 28 °C, 70% humidity, 200 rpm. After 24 h, 5 µL of growth medium (columns 2 and 7) was transferred to the four adjacent wells and another 24 h incubation was performed. 160 µL of SEM medium was added and the plates were incubated again under same conditions for 48 h. Laccase activity was measured as described previously. 

Second rescreening. Selected clones from first rescreening were incubated in 3 mL MM (Supplemented with glucose) at 28 °C, 200 rpm, 24 h. DH5α *E. coli* was transformed with plasmids isolated from MM cultures and *E. coli* cells grown overnight in LB + Ampicillin plates at 37 °C. Insolate plasmid from a single colony was obtained and sent to sequencing. After sequence confirmation, *S. cerevisiae* cells were transformed with confirmed plasmid. Five single colonies were picked and screened for each clone, as described above. 

Microfermentation of *S. cerevisiae* clones expressing the selected laccase variants for comparison studies were performed as described previously [39].

### 2.7. Flask Scale Production of Laccase Variants

*Saccharomyces cerevisiae* cells transformed with selected laccase genes were inoculated in 3 mL MM and incubated for 48 h, 200 rpm, 28 °C. An aliquot of the culture was used to inoculate 10 mL MM in 100 mL flasks (final optical density OD_600_ = 0.3) and incubated until OD_600_ around 1 was reached. Then, cells were diluted to OD_600_ = 0.1 in 30 mL EB medium in 100 mL flasks and incubated at 28 °C or 20 °C, 200 rpm. After maximum activity was reached, cells were centrifuged (5000 rpm, 4 °C) and supernatants concentrated with Amicon Ultra Centrifugal filters (30 KDa) at 5000 rpm, 10 min.

### 2.8. Laccase Characterization

Enzymatic assays were carried out in 96-well plates with 100 mU/mL of non-purified laccases (activity measured with ABTS at pH 3), in a SpectraMax M2 plate reader. The pH activity profiles, enzyme thermotolerance (T50, 10 min) and pH stability assays were, as previously described, measuring laccase activity in triplicate to determine mean values and standard deviations [39]. T50 (10 min) was defined as the temperature at which 50% initial activity is kept after 10 min incubation. Enzyme stability to the presence of solvents and halides was performed in triplicate as was described for pH stability assay [39], but using purified enzyme incubated in 20 mM Tris-HCl pH 7 with either Tween 20, CHAPS and PET detergents at 34 mM, NaCl at 2M or 0.1% SDS (*v/v*). 

Twenty μL aliquots of purified enzyme solution (at 100 mU/mL activity) were added to 170 μL 50 mM CP buffer (pH 3) in the presence of a gradient of 10% to 90% (*v/v*) of organic solvents (acetone, ethanol and DMSO) and different concentrations of halides (0.01–0.15 mM for NaF, 10–200 mM for NaCl, 0.1–15 mM for SDS and 5–200 mM for EDTA) (96-well plates). Ten μL ABTS 60 mM were added to the mixture (three replicates per sample) and laccase activity was immediately measured for 2 min. Solvent inhibition was fitted to a sigmoidal function and halides to a biexponential decay function to calculate IC50 values.

The activities of the crude enzymes were tested by quintuplicate in 96-well plates. The assays were performed by adding 20 μL of enzyme solution with 1 U/mL laccase activity (measured with ABTS at pH 3) to 180 μL of 3 mM DMP or 9 mM guaiacol in 100 mM acetate phosphate buffer (pH 5), to 180 μL of 5 mM DMPD or PPD in 100 mM tartrate buffer (pH 4) or to 180 μL of 100 µM EB in 100 mM tartrate buffer (pH 4). Respective activities were calculated by the increments in absorbance at 469 nm (εDMP = 27,500 M^−1^ cm^−1^), 470 nm (εguaiacol = 26,600 M^−1^ cm^−1^), 550 nm (εDMPD = 4134 M^−1^ cm^−1^) and 450 nm (εPPD = 14,685 M^−1^ cm^−1^). The specific activities (U/mg) of purified enzymes were calculated measuring the increment of absorbance at 409 nm (εHBT = 321 M^−1^ cm^−1^) in 100 mM acetate phosphate pH 5, and 410 nm (εaniline = 1167 M^−1^ cm^−1^), 515 nm (εvioluric acid = 113 M^−1^ cm^−^^1^, Figure S1) in 100 mM tartrate buffer pH 4. For EB and RB5, the activities were measured by the decrement in absorbance at 505 nm (εEB = 55,500 M^−1^ cm^−1^) and 495 nm RB5 (εRB5 = 22,500 M^−1^ cm^−1^) in 100 mM tartrate buffer pH 4. 

Kinetic constants were determined as previously described [42] using a range from 0.00125 mM to 0.1 mM for ABTS and DMP and 0.00125 mM to 2 mM for DMPD, and the following buffers: 100 mM CP buffer for assays at pH 3, 100 mM tartrate for pH 4 and 100 mM acetate phosphate for pH 5. To calculate *K*_M_ and *k_cat_* values the average Vmax was represented versus substrate concentration and fitted to a single rectangular hyperbola function in SigmaPlot (version 14.0) software for DMP and DMPD, and fitted to a Hill-sigmoidal for ABTS, where parameter a was equal to *k_cat_* and parameter b was equal to *K*_M_. In all assays three replicates of each laccase variant were used.

### 2.9. Purification of Laccase Variants

Crude enzymes were filtered with 0.22 µm pore size membrane and concentrated and ultra-diafiltrated using Pellicon cassettes (Merck Millipore, Darmstadt, Germany) and Amicon stirred cells (Merck Millipore, Darmstadt, Germany), both with a 10 kDa cutoff. The concentrated solution was dialyzed against 20 mM TrisHCl buffer pH 7 and immediately concentrated by Amicon Ultra (10 KDa) tubes at 5000 rpm, 10 min. Laccases were purified by FPLC (AKTA purifier system, GE Healthcare) in three steps: (i) anion exchange HiPrep QFF 16/10 column (GE Healthcare) using a salt gradient 0–40% (20 mM Tris-HCl, 1M NaCl, pH 7) to elute the enzyme; (ii) anion exchange Mono Q 5/50 GL column (GE Healthcare), using a 0–25% salt gradient (20 mM Tris-HCl, 1M NaCl, pH 7) to elute the laccase; (iii) molecular exclusion column HiLoad 16/600 Superdex 75pg (GE Healthcare). Between each purification step, fractions with laccase activity were collected, dialyzed and concentrated using Amicon^®^ Ultra (10 KDa) tubes. The purity of the enzyme was estimated in SDS-PAGE and *N*-deglycosylation was performed with Endoglycosidase H following manufacturer’s instructions. Additionally, a chromatographic affinity step with HiTrap Con A 4B column (GE Healthcare) was added for evaluating NGly laccases variants. The eluent buffer was 20 mM Tris-HCl, 0.5 M NaCl, 1M Glucose, pH 7.

### 2.10. Protein Quantification

Protein quantification was calculated by the Qubit 3.0 fluorometer of Sigma-Aldrich (St. Louis, MI, USA). 

## 3. Results

### 3.1. Selection of A. pediades Laccase

The fungus *A. pediades* is a saprotrophic Agaricales species representative of grass litter decomposers. The genome of *A. pediades* AH 40210 first sequenced in the framework of the multi-genome Joint Genome Institute (JGI) CSP15-1609 project was recently analysed in a comparative genomic study where the enzymatic lignocellulolytic machineries of 52 Basidiomycota (Agaricomycetes) species were evaluated. The fungus showed 13 MCOs: one ferroxidase, one novel laccase-ferroxidase and 11 sensu stricto laccases [23].

In this study, the fungus was grown on wheat straw and the inventory of secreted enzymes after 6, 14 and 43 days of solid-state fermentation was analysed and compared with the fungal secretomes obtained under non-ligninolytic conditions (liquid culture in glucose-ammonium medium) at the same incubation times. Proteins were identified by the presence of at least two unique peptides per protein (from tryptic digestion). The recurrent detection of peptides from a certain protein, indicated by the number of peptide spectral matches (PSM), was used for the semi-quantitative analysis of the abundance of the proteins identified in the secretomes. The production of only one out of the eleven laccases found in the genome was induced under ligninolytic conditions during the first days of solid-state fermentation (6 and 14-day samples), whereas the enzyme was not secreted under non-ligninolytic conditions (Table 1). This laccase, with ID 823363 JGI, from now on named as ApL (*A. pediades* laccase), was selected as the subject of this study, and its gene, more precisely its CDS, was synthetized de novo to be heterologously expressed. 

### 3.2. Heterologous Expression and Pre-Characterisation of Native ApL

*Saccharomyces cerevisiae* was transformed with the ApL CDS fused to the α_9H2_ leader, a signal peptide obtained in a previous laccase evolution campaign [20] that enhances the secretion of fungal laccases by the yeast [39]. The transformed cells were grown in flask liquid cultures for laccase expression and the secreted laccase activity monitored every 24 h by the oxidation of ABTS. Maximum activity (280 U/L) was reached after 4 d of fermentation (Figure S2A). The crude enzyme was pre-characterised. The optimal pH for oxidation of DMP was pH 4, and, although the peak of activity with ABTS was reached at pH 3, the enzyme showed around 20% activity with this substrate at pH 6 (Figure S2B), something unusual for a basidiomycete laccase [43,44,45]. The zymogram stained with DMP for detection of laccase activity showed a strong hyperglycosylation of native ApL (Figure S3).

### 3.3. Laccase Directed Evolution

**Figure 1:**
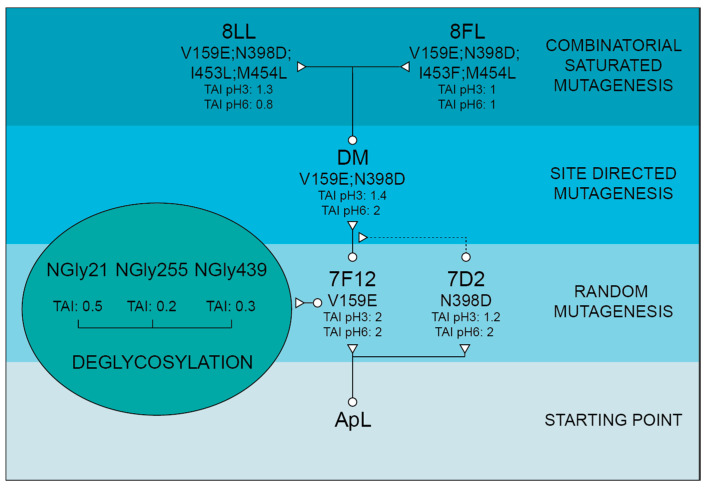
Evolution pathway of α_9H2_-ApL (from bottom to top). Three different approaches were combined to improve the heterologous production of the enzyme or its catalytic activity and shift of optimal pH: random mutagenesis over the whole construction, site-directed mutagenesis over 398th position and combinatorial saturation mutagenesis over 453rd and 454th positions. Besides, based on a consensus criterion, the effect of *N*-glycosylation on 7F12 variant was studied through site-directed mutagenesis of the three putative sites NGly21 (N21S), NGly255 (N256Q; T257P; S258V) and NGly439 (N439S). Mutations accumulated in ApL sequence in the successive engineering steps are shown. TAI indicates total activity improvements (produced either by enhanced laccase production and/or better catalytic activity) detected for the new variants compared to the activity of the laccase used as parent in each mutagenesis round (i.e. the fittest laccase of the previous generation).

Once ApL was functionally expressed by the yeast, α_9H2_-ApL construction was subjected to a round of random mutagenesis by epPCR and screening to facilitate the improvement of enzyme secretion, catalytic activity and/or shift of optimal pH. The mutant library was expressed in *S. cerevisiae* micro-fermentations, and the activities of the clones were explored by high-throughput screening with ABTS pH 3 and pH 6 (the latter was used to search for activity improvements at more neutral pH values). Two fittest variants were selected: 7F12, that exhibited a two-fold improvement in the laccase activity detected at both pH values and held V159E mutation, and 7D2, with twice better activity at pH 6 and 1.2-fold improvement at pH 3, that held the N398D mutation (Figure 1).

**Figure 2:**
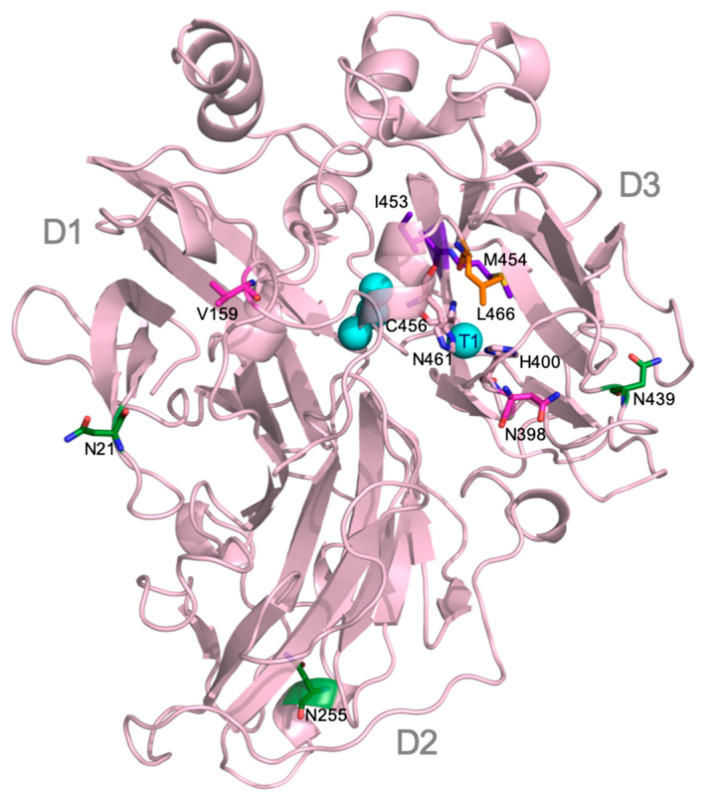
Structure model of native ApL represented as cartoon showing the typical folding in three cupredoxin-like domains (D1, D2 and D3), the four catalytic coppers (cyan spheres) and the two His and one Cys coordinating T1 copper (light pink sticks); residues mutated in this work are also shown as sticks: Val 159 and Asn 398 (in magenta), N-glycosylations sites Asn 21, 255 and 439 (in green); T1 “axial ligand” Leu 466 (in orange) and Ile 453 and M454 (in purple).

The two mutated residues are located far from each other in ApL 3D structure model (Figure 2). Both are placed in loops but while Val 159 is far from T1 site, Asn 398 is one of the residues delimiting the substrate-binding pocket [46]. To evaluate a possible additive effect between both mutations, N398D was introduced in 7F12 by site-directed mutagenesis obtaining the Double Mutated (DM) variant, with V159E and N398D. The three laccase variants DM, 7F12 and 7D2 were produced in yeast microfermentations and compared. DM exhibited better activity than 7F12 (1.4-fold) and 7D2 (2.6-fold) with ABTS at pH 3. Moreover, the activity at pH 6 was significantly improved (two-fold higher than 7F12), indicating a possible shifting of the pH activity profile (Figure 1). 

Next evolution cycle was focused on positions surrounding the conserved His-Cys-His tripeptide (HCH) involved in the intramolecular electron transfer between the T1 site and TNC [1]. The multiple sequence alignment of 482 Agaricales laccases available in JGI revealed variable amino acid frequency among the residues flanking the HCH tripeptide (Figure S4A). We randomized the most variable positions upstream (residues 453 and 454) and downstream (residues 459 and 460) from HCH motif in DM laccase variant. For that, two combinatorial saturation mutagenesis (CSM 453–454 and CSM 459–460) were performed using degenerate primers encoding the predominant amino acids found in the alignment (Figure S4B). After the screening, no clones from CSM 459–460 library could be selected with better or similar activities than the parent laccase, and the majority of clones from CSM 453–454 library also exhibited null or poorer activities than DM laccase, thus indicating an essential role of these position for laccase activity. However, we selected two new variants from the CSM 453–454 library. The 8LL variant (with two new mutations, I453L, M454L) exhibited a 1.3-fold TAI at pH 3 and lower activity at pH 6 (0.8-fold) than DM, whereas 8FL variant (I453F, M454L) showed parental-like activity at both pH values (Figure 1). Location of 453, 454 residues and other amino acid residues mutated in ApL are shown in Figure 2. 

**Figure 3:**
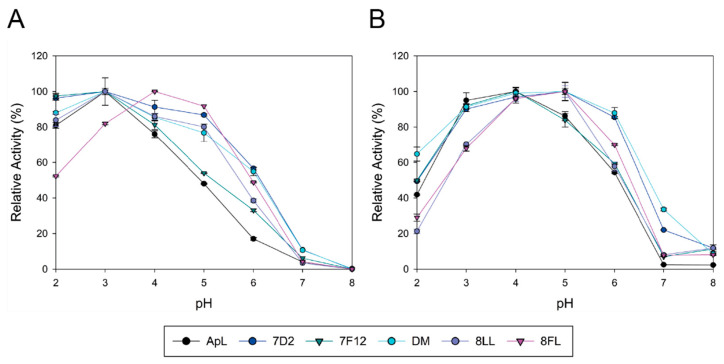
pH activity profiles of ApL and mutated variants for oxidation of ABTS (**A**) and DMP (**B**). Data are normalized to the maximum activity of each laccase.

Native ApL and its mutated variants were produced in *S. cerevisiae* in flask liquid cultures. After 96 h of fermentation at 28 °C, the OD_600_ of all yeast cultures were similar, but significant differences were found in the laccase activities detected in the liquid extracts (Table 2). The highest activity levels were found for the new variant 8LL followed by 8FL and DM, as observed in microcultures (Figure 1). The thermotolerance of crude laccases was evaluated by the T50 (10 min) assay. Variants holding mutation V159E (all except 7D2) showed a lower thermotolerance than native ApL. This decrease was stronger for CSM variants 8LL and 8FL, with decrements in T50 by 8 °C. Conversely, 7D2 kept the thermotolerance of native ApL (Table 2). In general, the mutated variants showed less acidic activity profiles, with improved activities at pH 4–6 (all kept over 80% activity at pH 5), except for 7F12 that maintained the same profile of native ApL (Figure 3A). All variants showed maximum activities with ABTS at pH 3, except for 8FL, with maximum activity at pH 4 and a notable shifted profile to more neutral values. As regards DMP oxidation, ApL showed maximum activity at pH 4, that was shifted to pH 5 in all mutated variants, except for 7F12 (with optimal pH 4). Moreover, 7D2 and DM laccases retained over 90% activity at pH 6 and 20% at pH 7 (Figure 3B).

We next evaluated the oxidation capabilities of all laccase variants for the oxidation of different model compounds, including phenols (DMP and guaiacol) and aromatic amines (DMPD and PPD) (Table 3). In order to better compare the oxidation abilities of the different crude enzymes, all reactions were carried out with 100 mU/mL of each laccase (activity measured with ABTS pH 3). Compared to ABTS, all laccase variants oxidised phenolic compounds worse and DMPD better. Variants 7D2 and DM were, on average, better for phenol oxidation than the others, 7D2, in particular with DMP, and DM with guaiacol. Variants 7D2, DM and 8FL were also better for DMPD oxidation, whereas very similar activities with PPD were observed in all laccase variants. 

Since DM variant combines high secreted activity levels, good thermotolerance, proper oxidation of different substrates and a broadened pH activity profile, it was selected for further assays, the results of which are shown below.

### 3.4. Exploration of Axial Ligand 

In the majority of characterised HRPLs from Polyporales a noncoordinating Phe residue occupies the position of Met that acts as the fourth axial ligand in low-redox potential laccases from plants and bacteria. In ApL this position is occupied by a Leu (Leu 466). We studied the impact of the amino acid located in position 466th by saturation mutagenesis in DM laccase. During the screening of the library, only laccase variants with a Leu (DM as such), Phe (DM-Phe) or Met (DM-Met) in this position exhibited detectable laccase activity, mimicking the natural restriction to the same three amino acids in laccases. Then, DM-Phe and DM-Met variants were produced in flask and their oxidation capabilities compared with those of DM under same conditions (Table 2). A modest increase in activity with ABTS (1.2-fold) was observed for DM-Phe, and 0.8-fold diminished activity for DM-Met. Besides, DM-Phe and DM variants showed same T50, while thermotolerance of DM-Met seemed to be slightly lower (1 °C) (Table 2). 

**Figure 4:**
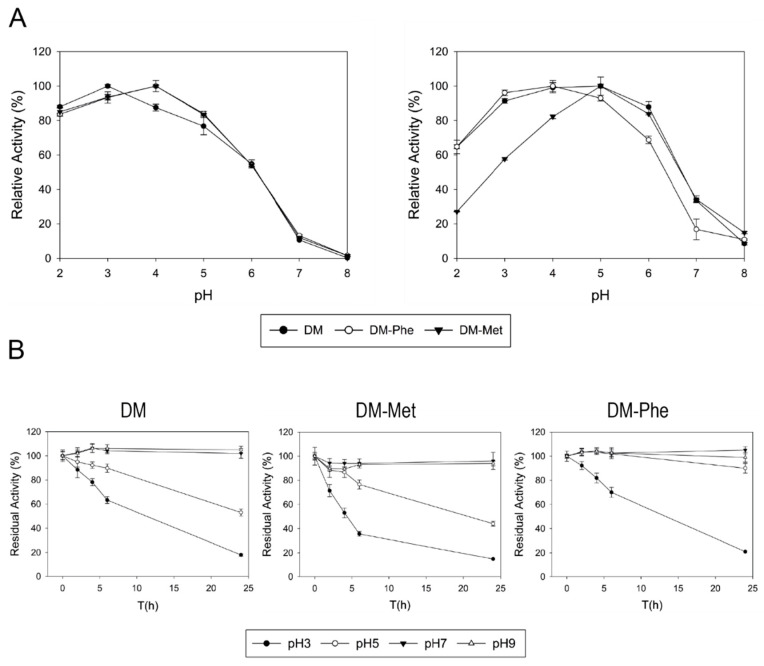
(**A**) pH activity profiles of DM and axial-ligand variants (DM-Phe and DM-Met) for the oxidation of ABTS (**left**) and DMP (**right**). (**B**) pH stabilities of, from left to right, DM, DM-Met and DM-Phe.

The three laccase variants exhibited quite similar pH activity profiles for oxidation of ABTS, whereas significant differences were observed for the oxidation of DMP (Figure 4A). DM-Met exhibited a narrowed activity profile than DM with diminished activity at pH < 5, but maintained its maximum activity at pH 5 and similar profile to DM at pH > 5. Conversely, the DM-Phe variant showed a more acidic profile, with maximum activity at pH 4 and reduced activity at pH > 4. All variants had notable stability at neutral and basic conditions, but acid pH seemed to destabilize the enzymes. Surprisingly, DM-Phe was the only variant able to maintain almost 100% activity after 24 h at pH 5 (Figure 4B). 

In order to check any possible variation in the oxidative capabilities of DM due to the replacement of Leu by Met or Phe, we repeated the assay with the two axial-ligand mutated variants for the oxidation of different model compounds, using 100 mU/mL of laccase activity with ABTS. On top of the aforementioned compounds, we included the Evans Blue dye as a substrate recalcitrant to oxidation for comparison of DM variants. Except for the better oxidation of DMP, the oxidative capabilities of DM-Met were significantly inferior, whereas DM-Phe did not improve the oxidation of any substrate with respect to DM laccase (Table 3). 

### 3.5. Enzyme Kinetics

DM was purified and further characterised as the most relevant ApL variant. Its kinetic constants for the oxidation of ABTS, DMP and DMPD were determined and compared with those of the 7F12 variant (Table 4). The latter was used as reference of the native ApL because 7F12 variant was three times better produced but it maintained the same oxidation capabilities than the native enzyme, differing only in one amino acid (V159E mutation) located in an external loop. Furthermore, since DM and 7F12 variants only differ in mutation N398D, it allowed us to explore the contribution of this amino acid substitution (located in the binding pocket) to the performance of the enzyme. DM variant showed superior catalytic efficiencies than 7F12 for all substrates due to the 10 to 20-fold improvement in *k*_cat_ (the catalytic efficiency of DM for oxidation of DMPD is outstanding). The improved *k*_cat_ values compensated for the lower affinities for DMPD and DMP while, in combination with the high affinity for ABTS, they raised the catalytic efficiency for the oxidation of this substrate to remarkable values in the DM variant. 

We then calculated the specific activity of DM laccase towards two recalcitrant industrial dyes (EB and RB5), two high-redox potential mediator compounds (violuric acid and HBT) and another aromatic amine (aniline). In this assay we used PM1L as a reference of a Polyporales laccase with high redox potential [47]. Like PM1L, DM was able to oxidise the five substrates, and its activity on violuric acid outperformed 10 times that of PM1L (Table 5).

Several organic solvents, halides or denaturing agents were investigated as inhibitors of DM laccase. By using the IC50 assay we calculated the concentration of the substance required to inhibit by 50% the activity of the enzyme. DM showed good activity in the presence of organic solvents and EDTA and variable behaviour in the presence of halides (Table 6). Furthermore, the stability of the developed laccase was evaluated by incubating the enzyme with different organic solvents, halides and detergents for 24 h (Figure S5). In general, DM laccase showed a notable stability towards long term exposure to 34 mM CHAPS and 34 mM PET and maintained 70% of the initial activity after 24 h in 0.1% SDS. The purified enzyme also retained notable activity after 24 h in presence of the halide (2M NaCl), suffering a larger destabilization in the presence of 60% organic solvents (Ethanol and DMSO).

### 3.6. N-glycosylation Studies

Preliminary characterization of ApL revealed a strong hyperglycosylation of the enzyme. This was confirmed by the smear (from above 250 KDa to 100 KDa) observed in the SDS-PAGE of the purified 7F12 variant (Figure S6). After deglycosylation with Endo H, the enzyme recovered the theorical MW of 55 KDa for ApL. Three most probable *N*-glycosylation sites, N21, N255 and N439, were predicted in ApL by the NetNGlyc 1.0 Server according to the Asn-X-Ser/Thr sequence (where X is any amino acid except for Pro). In order to check the contribution of *N*-glycosylation to the catalytic activity or secretion of laccase, the three putative sites were individually removed in 7F12, producing the respectively mutated NGly21, NGly255 and NGly439 laccase variants. The target residues were substituted by the most frequent amino acid residues found in these positions in the multiple alignment of 482 sequences of Agaricales laccases (Figure S7). The evaluation of the *N*-glycosylation sites was carried out on 7F12 variant because, as aforementioned, it was three times better produced than native ApL (due to the amino acid substitution V159E in an external protein loop), as evidenced by the same activities of both enzymes for different substrates.

The three *N*-glycosylation mutants and 7F12 laccase were produced in *S. cerevisiae* in flask liquid cultures at two different temperatures. Maxima laccase activities were reached in the culture broths after 96 h and 120 h for 28 °C and 20 °C cultures, respectively. All laccases were better produced at 20 °C, although significant lower activity levels were detected for the NGly variants, especially for NGly255 and NGly439 variants. These differences were even more pronounced at 28 °C (Table 7). 

**Figure 5:**
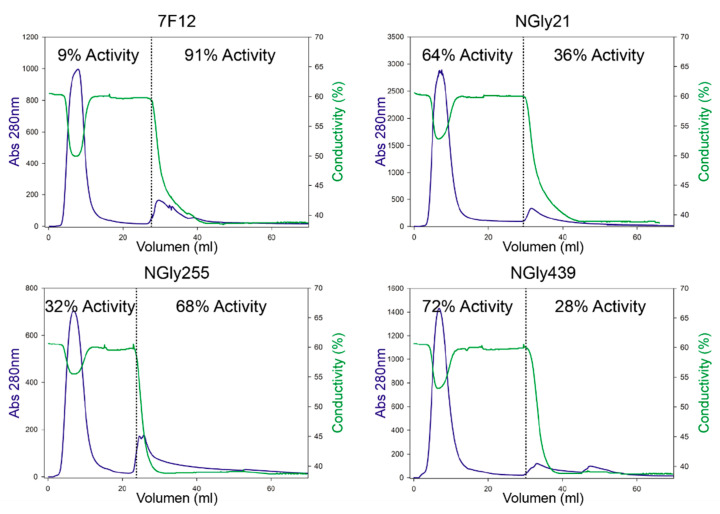
Concanavalin A affinity chromatography of the native glycosylated 7F12 laccase and its NGly21, NGly255 and NGly439 variants. The different *N*-glycosylation patterns are indicated by the percentages of laccase activity found in the non-retained fraction (**left**) and in the retained-glycosylated-fraction (**right**) in each variant. Protein profile was monitored by A280 nm (blue); the drop in conductivity (green) indicates the change to elution buffer to recover the glycosylated form retained in the column.

**Figure 6:**
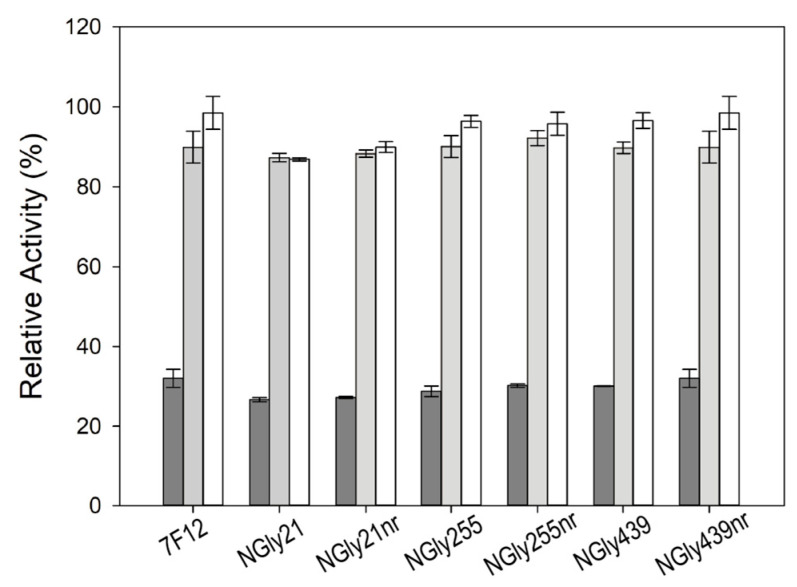
pH stability of parent laccase (7F12) and its NGly variants separated into their retained and non-retained (nr) fractions; Assay was carried out at pH 5 (dark grey bars), 7 (light grey bars) and 9 (white bars); residual activities after 24 h were measured with ABTS pH 3.

Laccase 7F12 and its NGly variants were purified from the culture broths of 20 °C fermentations. After two anionic exchange chromatography steps, an aliquot of every partially purified laccase was subjected to a concanavalin A affinity chromatography to evaluate enzyme glycosylation. This chromatography step enables separation of glycosylated and non-glycosylated isoforms. Laccase activity was measured with ABTS pH 3 in the fractions retained in the column (glycosylated) and in the non-retained (non-glycosylated) ones (Figure 5). The majority (91%) of 7F12 laccase activity was detected in the retained fraction, in concordance with the strong glycosylation of the enzyme. By contrast, only 68% of NGly255 activity was retained in the column, and up to 64% and 72% of respectively NGly21 and NGly439 laccases were not retained in the column, suggesting that the removal of the *N*-glycosylation sites led to notably less glycosylated variants (Figure 5). SDS-PAGE of all retained and non-retained fractions (Figure S8) added evidence about the real (not putative) contribution of the three sites to the glycosylation of the enzyme. We evaluated the thermotolerance and stability to pH of the retained and non-retained fractions of 7F12 laccase and NGly variants. No significant differences were observed in T50 (10 min) values (Table S2). Nor was a correlation found between enzyme stability to pH and glycosylation (Figure 6).

Thereafter, to evaluate the kinetics constants of 7F12 laccase and NGly variants, we completed the purification of the enzymes by applying an exclusion chromatography step onto partially purified enzymatic aliquots prior to the concanavalin A affinity chromatography step (in order to avoid that the harsh conditions of the latter could have spoiled laccase activity). The kinetic constants for the oxidation of ABTS and DMP are shown in Table 8. The NGly variants showed reduced catalytic efficiencies for the oxidation of both substrates as compared with 7F12 laccase due to diminished catalytic constants, being that this effect more pronounced in NGly21 and NGly255 variants. The drop in catalytic efficiency was stronger for ABTS due to the lower affinity of NGly variants for this substrate (equal for the three variants), whereas affinities for DMP were slightly increased in the NGly variants. 

## 4. Discussion

*Agrocybe pediades* is a representative Agaricales species that grows on pastures and meadows (grass-litter lifestyle). Although its genome encodes 11 different laccases, all classified as sensu stricto [23], only the laccase named ApL in this study was secreted by the fungus under ligninolytic conditions (solid-state fermentation of wheat straw). We addressed the functional expression, engineering and characterization of this enzyme using *S. cerevisiae* as a heterologous expression system. The yeast was chosen since it is the preferred platform for the directed evolution of fungal laccases and has provided successful secretion of active enzymes with interesting properties as biocatalysts [20,32,47].

In a previous work, we demonstrated the capability of the evolved α_9H2_ leader [20] to improve the secretion by *S. cerevisiae* of several fungal laccases (ApL included), compared to different signal peptides derived from the α-factor preproleader [39]. Here, the use of α_9H2_ leader allowed us to achieve the functional expression of ApL in the yeast, although the levels obtained were insufficient for a deep characterization of the enzyme. To increase laccase production, and simultaneously to give rise to an improved version of the enzyme as a biocatalyst, the laccase was first subjected to a round of directed evolution through random mutagenesis over the α_9H2_-ApL construction. Mutation V159E selected in 7F12 variant produced two-fold TAI in microfermentation, without affecting the optimum pH of the enzyme. The location of V159E mutation at the protein surface, far away from the T1 site (Figure 2), together with the similar activities for different substrates of native ApL and 7F12 variant, evidenced an enhancement of enzyme production by this mutation. Analysis of the structure model of ApL suggested that V159E substitution caused no changes in H bonding or electrostatic interactions (using the evaluating salt bridge server ESBRI [38]). Although it is not possible to conclude what the exact contribution of this mutation is, it might facilitate the polypeptide maturation during expression and secretion by the yeast, in agreement with reported single-point mutations enhancing protein expression by aiding in protein flexibility during folding [32] or stabilizing buried regions [48].

Basidiomycete laccases generally exhibit acidic pH activity profiles [44,49]. By contrast, ApL shows significant activity at pH 6. This constitutes an interesting property that can be enhanced in the laboratory to facilitate the applicability of the enzyme since alkaline conditions are required for industrial applications such as lignin valorization [50,51]. Thus, we included an activity assay at pH 6 during the screening of the mutant library to facilitate the selection of mutations enhancing the activity of the enzyme at neutral pH. As a result, 7D2 variant, holding mutation N398D, was selected with improved activity at pH 6. Based on these results and on the distance between V159E and N398D, we evaluated their possible joint effect in the DM variant (V159E; N398D). DM exhibited properties from their parents: it showed a wider pH profile towards neutral pH and notably higher laccase activity with respect to native ApL. Then, we studied the amino acids adjacent to the conserved tripeptide His-Cys-His. These residues coordinate T3 and T1 coppers, and the Cys-T1 bond has a strong influence on laccase activity [1]. The alignment of 482 laccase sequences of 33 fungal Agaricales genomes revealed differences in conservation among the residues upstream and downstream the tripeptide. Therefore, the variable positions were randomised in the DM variant in two independent mutagenesis libraries, CSM 453–454 and CSM 459–460, using customised primers to substitute the residues of ApL by the most frequent amino acids found in the alignment. Noticeably, in the CSM 459–460 library, the pair Asp 459, Trp 460 of DM laccase was the only combination that ensured detectable laccase activity, suggesting a strong restriction in these positions for ApL (even amino acid changes of same nature are not allowed). In CSM 453–454 only two new amino acid combinations provided parental-like activities: I453L, M454L (8LL variant) and I453F, M454L (8FL variant). Mutations selected in 453 position were conservative (Ile was replaced by Leu or Phe), suggesting that a hydrophobic residue is required in this position, whereas in both mutants Met 454 was replaced by Leu. Important changes in the pH activity profiles (significant narrower and more neutral profiles with ABTS and DMP for variant 8FL) and in T50 (significantly diminished in 8LL and 8FL) indicated that the amino acids placed in these positions not only affect the pH dependence of laccase activity, but also the thermotolerance of the enzyme. Contribution of the second-shell amino acids of T1 copper to modulate laccase activity and restriction to acid pH in fungal HRPLs has been previously reported [52,53]. 

Substitution of Asn 398 by Asp stood out as the most relevant mutation for the catalytic activity of the enzyme. Asn 398 is located in a loop delimiting the entrance of the substrate binding pocket, nearby His 400 (T1 Cu ligand). Comparison of 7F12 and DM catalytic constants for the oxidation of different model compounds showed that N398D mutation is responsible for a notable superior catalytic efficiency of DM and a significant improvement of activity at neutral pH values. This is in agreement with previous results found in Ascomycete [52] and Basidiomycete [32,42] laccases where substitution in positions equivalent or contiguous to ApL 398th produced an increment in laccase activity and a shifted profile to more neutral pH values. Actually, P394H substitution (equivalent to 399 ApL) increased the activity at neutral pHs of a swap-domain laccase [42], shifted the optimal pH from 3 to 5 (with DMP) and improved the catalytic activity of an evolved *Pycnoporus cinnabarinus* laccase [54]. In the latter enzyme a new substrate binding mode increases the turnover rate associated to an enhanced stabilization of the oxidized form of certain substrates [55].

On the other hand, the kinetic parameters of DM variant for the oxidation of model compounds are remarkable. Its catalytic efficiencies for the oxidation of ABTS and DMP are notably superior than those of other wild laccases from Agaricales like *Agaricus blazei* laccase [56] or HRPLs from Polyporales such as PM1L [13] or *Trametes trogii* laccase [10]. In addition, the kinetic constants of DM with these two substrates are also notably superior to those of laccases from *Coprinopsis cinerea* [57] and *Pleurotus sajor-caju* [58] or *Trametes versicolor* [53] expressed in yeast. Moreover, the specific activities of the developed ApL for the oxidation of recalcitrant organic dyes or high-redox potential mediators is similar or better than those of PM1L, suggesting the enzyme possesses a high redox potential as well, although it holds a Leu as fourth “non-coordinating” axial ligand instead of a Phe typical of Polyporales HRPLs. In addition, the activity of the DM variant was not severely affected by the presence of the different inhibitors tested here. In fact, the enzyme showed high tolerance to the presence of EDTA, SDS and halides, with IC50 superior to those of other fungal laccases [59]. The enzyme exhibited a striking activity under the presence of EDTA and organic solvents by contrast to other laccases, which in general hardly tolerate high concentration of these inhibitors [12,60,61,62]. It also exhibited outstanding stability to NaCl, whereas the tolerance to NaF was remarkably poorer. These results are in agreement with the reported strong potential of fluoride to obstruct the electron transfer in the TNC site, due to the influence that the diameter of the anion has on the inhibitory potential of the halide (Fl- > Cl- > Br-) [19,60,63].

A Phe residue is fully conserved as the fourth non-coordinating axial ligand of T1 in the majority of the HRPLs crystalized so far (most of them from Polyporales). Some examples with Phe in this position are those from *Trametes versicolor* [7], *Trametes trogii* [64], *Coriolopsis caperata* [65], *P. cinnabarinus*, PcL [66] or PM1 basidiomycete [13,18]. Instead, ApL has a leucine as non-coordinating residue in the axial position. The correlation between the high redox potential of the T1 site and the presence of Phe at this position has been called into question because other factors like the charge distribution near T1 site [67] or the length of the bonding between T1 and its ligands [7] seem to exert an effect on the redox potential of laccases. In this line, a laccase from *Rigidosporus lignosus* exhibited a 730 mV with a Leu [68] or, more noticeable, an Ascomycete laccase from *Botrytis aclada* with a Leu at this position also showed a considerable redox potential (720 mV) [69]. Here we proved the oxidation of several recalcitrant substrates by DM, suggesting that this ApL variant possesses a high-redox potential comparable to that of the HRPL from PM1 basidiomycete. DM showed even superior specific activity than PM1L with violuric acid and RB5, although these differences could be attributed to substrate affinity that is determined by the size, shape or polarity of the substrate binding pocket [18,70]. The oxidation of the high-redox mediator violuric acid and the organic dye RB5 are of relevance for the respectively application of the enzyme in laccase-mediator systems and in the degradation of industrial dyes [15,17]. Saturation mutagenesis of this position in DM variant confirmed the preference of Leu in ApL scaffold. Substitution by Met barely allowed the oxidation of guaiacol or EB dye by DM-Met variant, probably related to a decrease in the redox potential accordingly to other reports [69]. On the other hand, while DM-Phe variant exhibited better activity with ABTS than DM, it also showed poorer oxidation for EB dye or guaiacol. Even though these differences could be attributed to the strong dependence of pH profiles on the axial ligand shown here and in other studies [52], this does not seem to determine the oxidation of EB dye, guaiacol or PPD because the assays were carried out at pH values where both enzymes work efficiently. 

Glycosylation is a major post-translational modification described to facilitate protein folding and structural stabilization or prevent from protein proteolysis [28,29,71]. Most fungal laccases are glycoproteins with glycan moieties contributing to up to 25–50% of the enzyme molecular weight [25,32,42,72]. *N*-glycosylation is known as the predominant type of sugar anchoring in fungal laccases obtained by either homologous [7,65,73] or heterologous [42,74,75] expression. The *N*-glycosylation patterns of Basidiomycete [74,76,77] and Ascomycete [75] laccases are clearly different. Most of these studies provide a reliable position of the sugar residues because they are based on crystallized structures, but the number of sampled laccases is still reduced, in particular of Agaricales laccases (only one crystallized [78]). Taking all this into account, we compared the three *N*-glycosylation sites of ApL (N21, N255 and N439), each respectively placed in one of the three cupredoxin domains of laccase (D1, D2 and D3) (Figure 2), with those predicted by the NetNGlyc 1.0 Server in laccases from Polypolares (82 laccases with PcL as query sequence) and from Agaricales (482 laccases with ApL as query sequence). In Polyporales laccases, two sites, N54 and N434 (PcL numbering), located in D1 and D3, respectively, were almost fully conserved [74,76,77]. On the contrary, Agaricales laccases exhibited more variability of *N*-sites among the three laccase domains, although N434 (N439 in ApL numbering) stood out as the most conserved *N*-glycosylation site (92% frequency). The three putative *N*-glycosylation sites of ApL were later proved as real sugar anchoring sites after we individually removed the three sites and the corresponding N-Gly laccase variants were subjected to concanavalin A chromatography. This allowed us to detect the partial deglycosylation of the three NGly variants, that showed dissimilar glycosylation patterns among them and different from the full glycosylated enzyme (7F12) holding the three sugar-anchoring sites. 

To study if *N*-glycosylation in these sites is critical for the heterologous expression and activity of ApL, the three *N*-Gly variants were produced in *S. cerevisiae* flask cultures and characterised. All *N*-Gly variants showed diminished laccase activities in the crude extracts, especially those removing N255 and N439 sites, which barely reached a 12% and 5% of the detected parental activity. These results confirm the crucial role of both sites. The essential role of N439 had been suggested by the strict conservation of this *N*-glycosylation site in Agaricales and Polyporales laccases. Besides, our results agree with those obtained with *Lentinus sp* laccase where almost no activity was found when N238 and N458 sites (coinciding with N255 and N439 in ApL) were removed [74]. The role of sugar anchoring at these positions was proposed as a mechanism to maintain the intrinsic laccase activity by stabilizing a large loop connecting the two cupredoxin domains D2–D3 [49,74]. Some authors reported that the lack of sugar could derived into conformational changes for substrate binding during the catalytic reaction, which is supported by the correlations found between deglycosylation of laccase and loss of activity [73,79,80]. However, these studies were based on the kinetic characterization of the recombinant enzymes once secreted by the yeast and after enzymatic deglycosylation, without considering the effect that *N*-glycosylation may have in early protein processing (post-translational modification, folding, secretion, etc.). Aiming at evaluating the biological role of glycosylation on the heterologous expression of ApL and/or on its catalytic activity, we purified the three *N*-Gly variants specifically obtained from the removal of each site N21, N255 and N439. Comparison of their kinetic constants showed, in general, poorer activities than the parent laccase (7F12 used as reference for native glycosylated laccase) due to lower turnover rates for the oxidation of ABTS and DMP. However, this does not fully explain the outstanding decrease of activity detected in the corresponding *S. cerevisiae* liquid cultures, specially at 28 °C. In fact, reduction of the temperature to 20 °C (to maximize the synthesis of correctly folded heterologous protein) raised the laccase activities detected in the culture broths and shortened the differences among *N*-Gly variants and parent laccase. The latter could be attributed to an increment on enzyme secretion by the yeast [81], avoiding harmful protein aggregation [82]. All these pieces of information pointed out to a possible double function of *N*-glycosylation in ApL, having an effect on enzyme production and activity. To evaluate both contributions, for each NGly variant and respecting the parent laccase, we compared the decrease in activity detected during 20 °C yeast fermentation and the decrease in *k*_cat_ for ABTS oxidation (under saturated substrate conditions the catalytic activity depends on this parameter). In the case of NGly21 variant both decrements were similar (around two-fold), indicating the contribution of glycosylation on N21 site only to enzyme activity. Conversely, the effect of sugars linked on N255 and N439 sites seemed to exert an effect on the catalytic activity (impaired two-fold and 1.3-fold, respectively) and, particularly, on the production of the enzyme by the yeast (the activities secreted in the culture broths were reduced by six- fold and 3.1-fold, respectively).

Finally, on the basis that glycoproteins with a similar degree of glycosylation could have different stability depending on where the sugar are linked [28], we studied the stability to pH and temperature of the different (de)glycosylated fractions of the NGly variants. No major differences were found among the stability of NGly variants and parent laccase, showing no direct correlation between the glycosylation degree and the thermotolerance or stability to pHs of ApL. These data disagree with the commonly accepted role of glycosylation to enhance enzyme thermostability [83] but are in agreement with other studies on fungal laccases where deglycosylation of the enzyme did not alter thermostability [42,74]. 

## 5. Conclusions

In this study a novel fungal laccase secreted by *A. pediades* under ligninolytic conditions was synthesised de novo and successfully expressed in *S. cerevisiae* using an evolved α-factor preproleader and enzyme directed evolution. Like other laccases from soil inhabiting Agaricales, the enzyme shows a more neutral pH activity profile than laccases from white-rot Polyporales, which show optimal acid pH in accordance with the pH of lignin degradation during wood decay. The mutagenesis of ApL and the characterization of the new variants allowed us to provide new insights on laccase structure-function, proving the contribution of certain residues of T1 environment to modulate laccase catalytic activity or to modify the optimal pH, and of residues located in the protein surface to raise laccase production. In addition, the selected new variant, with only two mutations of difference respecting native ApL, shows interesting properties as a biocatalyst similar or even better than HRPLs from Polypolares. It possesses improved activity at neutral pH and notable catalytic efficiencies oxidising model substrates, its tolerance to different inhibitors is remarkable, and it is capable to oxidize high-redox potential mediator compounds and recalcitrant organic dyes, suggesting a high redox potential for the enzyme. Finally, we demonstrate the *N*-glycosylation of the laccase in three predicted sites. The removal of these sites by site-directed mutagenesis and the characterisation of the partially deglycosylated variants, allowed us to unveil, for the first time, the impact that glycosylation in each particular site has on laccase secretion by the yeast and on its catalytic activity. 

## Figures and Tables

**Table 1:** Analysis of MCOs found in the secretomes of *A. pediades* grown under ligninolytic conditions (solid-state fermentation on wheat straw) and non-ligninolytic conditions (liquid culture in glucose-ammonium medium). Laccase (LAC), ferroxidase (FOX) and novel laccase-ferroxidase (NLAC-FOX). Out of the set of proteins identified in the fungal secretome after 6 d (236 proteins) or 14 d (192 proteins) of solid-state fermentation, laccase ID 823363 occupied the 132nd and 161st positions, respectively, in terms of PSM values. The majority of MCOs were not detected (-).

		Wheat Straw			Liquid Culture		
MCO Type	Protein ID (JGI)	6 d	14 d	43 d	6 d	14 d	43 d
LAC	639182	-	-	-	-	-	97/242
LAC	708834	-	-	-	-	-	-
LAC	709683	-	-	-	-	-	-
LAC	760749	-	-	-	-	-	-
LAC	734797	-	-	-	-	-	-
LAC	725377	-	-	-	-	-	-
LAC	741811	-	-	-	-	-	-
LAC	744557	-	-	-	-	-	-
LAC	823363	132/236	161/192	-	-	-	-
LAC	816500	-	-	-	-	-	-
LAC	816391	-	-	-	-	-	-
FOX	657643	144/236	-	-	-	-	-
NLAC-FOX	821086	-	-	-	-	-	-

**Table 2:** Secreted laccase activities and OD_600_ of flask liquid cultures of *S. cerevisiae* expressing the native ApL and the developed variants after 96 h of incubation at 28 °C. Thermotolerance is presented as T50 (10 min) values.

Laccase	ABTS (U/L)	OD_600_	T50 (°C)
ApL	135 ± 25	31 ± 1	69.9 ± 0.4
7D2	348 ± 10	34 ± 2	68.8 ± 0.6
7F12	425 ± 8	35 ± 1	65.8 ± 0.6
DM	608 ± 25	34 ± 1	65.8 ± 0.4
8LL	781 ± 30	35 ± 2	62.5 ± 0.4
8FL	620 ± 11	34 ± 2	61.7 ± 0.3
DM-Phe	708 ± 19	30 ± 1	65.4 ± 0.2
DM-Met	505 ± 14	32 ± 1	64.3 ± 0.3

**Table 3:** Activities of native ApL and its developed variants for the oxidation of phenols, aromatic amines and EB dye relative to the activity with ABTS (same activity units—100 mU/mL—of each enzyme with ABTS added in all assays); n.d.: non determined.

Laccase	DMP	Guaiacol	DMPD	PPD	EB Dye
ApL	34 ± 1	4.5 ± 0.2	165 ± 4	16 ± 0.1	n.d.
7D2	63 ± 5	6.1 ± 0.1	186 ± 3	20 ± 0.6	n.d.
7F12	32 ± 1	4.9 ± 0.1	150 ± 3	18 ± 0.4	n.d.
DM	44 ± 1	9.2 ± 0.1	194 ± 2	18 ± 0.7	1.5 ± 0.01
8LL	20 ± 1	3.2 ± 0.3	163 ± 2	17 ± 0.5	n.d.
8FL	38 ± 4	5.2 ± 0.2	179 ± 1	16 ± 0.9	n.d.
DM-Phe	47 ± 4	4.4 ± 0.2	197 ± 2	15 ± 0.5	0.6 ± 0.03
DM-Met	50 ± 2	0.4 ± 0.04	158 ± 7	9 ± 0.7	0.3 ± 0.01

**Table 4:** Kinetic constants for oxidation of model substrates by 7F12 and DM laccase variants.

	7F12			DM		
	*k*_cat_ (s^−1^)	*K*_M_ (mM)	*k*_cat_ /*K*_M_ (s^−1^ mM^−1^)	*k*_cat_ (s^−1^)	*K*_M_ (mM)	*k*_cat_ /*K*_M_ (s^−1^ mM^−1^)
ABTS pH 3	33 ± 1	0.0045 ± 0.0002	7391 ± 346	347 ± 0.8	0.0046 ± 0.0003	75500 ± 6138
DMP pH 5	6 ± 0.1	0.016 ± 0.001	371 ± 30	120 ± 2	0.08 ± 0.005	1490 ± 33
DMPD pH 4	56 ± 3	0.53 ± 0.05	107 ± 10	641 ± 17	1.7 ± 0.1	388 ± 65

**Table 5:** Specific activities (U/mg) of DM laccase for the oxidation of aniline, RB5 and EB dyes, and violuric acid and HBT mediators.

	Aniline	RB5 Dye	EB Dye	HBT	Violuric Acid
DM	18 ± 1	4.4 ± 0.4	8.6 ± 0.1	2.2 ± 0.3	0.02 ± 0.001
PM1L	27 ± 3	2.4 ± 0.3	22 ± 0.1	7 ± 0.6	0.003 ± 0.0002

**Table 6:** Inhibition (IC50 assay) of purified DM laccase by different substances.

	IC50 (mM or v/v % ^a^)
Ethanol ^a^	73 ± 1
Acetone ^a^	55 ± 2
DMSO ^a^	58 ± 1
NaCl	74 ± 1
EDTA	90 ± 3
NaF	0.051 ± 0.002
SDS	0.7 ± 0.05

“^a^” refers to the percentage (volume/volume) of organic solvent in the solution as it is indicated in the table: *v/v* %.

**Table 7:** Secreted laccase activities and OD_600_ of flask liquid cultures of *S. cerevisiae* expressing 7F12 laccase and its *N*-glycosylation mutants at different temperatures (28 °C and 20 °C).

Laccase	ABTS (U/L)	T (°C)	Time (h)	OD_600_
7F12	611 ± 13	28	96	31 ± 2
NGly21	335 ± 43	28	96	32 ± 2
NGly255	33 ± 3	28	96	34 ± 1
NGly436	73 ± 6	28	96	36 ± 2
7F12	778 ± 3	20	120	29 ± 0.1
NGly21	400 ± 11	20	120	27 ± 1
NGly255	133 ± 3	20	120	30 ± 1
NGly436	253 ± 20	20	120	30 ± 2

**Table 8:** Kinetic constants of 7F12 laccase and its NGly variants.

	ABTS pH 3			DMP pH 5		
	*K*_M_ (mM)	*k*_cat_ (s^−1^)	*k*_cat_ /*K*_M_ (s^−1^ mM^−1^)	*K*_M_ (mM)	*k*_cat_ (s^−1^)	*k*_cat_ /*K*_M_ (s^−1^ mM^−1^)
7F12	0.0045 ± 0.0002	33.26 ± 0.5	7391 ± 346	0.016 ± 0.001	6 ± 0.1	371 ± 30
NGly21	0.0051 ± 0.0002	14.77 ± 0.24	2896 ± 123	0.012 ± 0.001	2.27 ± 0.04	183 ± 12
NGly255	0.0052 ± 0.0002	15.21 ± 0.21	2926 ± 120	0.012 ± 0.002	2.38 ± 0.07	202 ± 26
NGly439	0.0051 ± 0.0002	25.95 ± 0.4	5088 ± 214	0.011 ± 0.0013	3.36 ± 0.07	305 ± 37

## Data Availability

Not applicable.

## Supplementary Materials

The following are available online at https://www.mdpi.com/article/10.3390/jof7050359/s1, Figure S1: Mass extinction coefficient of violuric acid, Figure S2: Time course analysis of ApL activity secreted by *S. cerevisiae* and pH activity profiles, Figure S3: SDS-PAGE of crude ApL, Figure S4: Combinatorial saturation mutagenesis on 453 and 454 or 459 and 460 residues in DM laccase variant, Figure S5: Stability of purified 7F12 variant to the presence of different substances, Figure S6: SDS-PAGE of 7F12 before and after deglycosylation with Endo H, Figure S7: Sequence logos for the three putative *N*-glycosylation sites (N21, N255 and N439), Figure S8: SDS-PAGE of 7F12 laccase and its NGly variants, Table S1: Sequences of the primers used, Table S2: Thermal stabilities of laccase NGly variants as T50 (10 min) assay.
