# Supplementary material for: Heterologous Expression, Engineering and Characterization of a Novel Laccase of Agrocybe pediades with Promising Properties as Biocatalyst
Source: J Fungi (Basel). 2021 May 4;7(5):359. doi: 10.3390/jof7050359 (PMC8147764; doi:10.3390/jof7050359)
Supplement: Supplementary file 1 [file jof-07-00359-s001.zip › jof-1182368-supplementary.pdf]

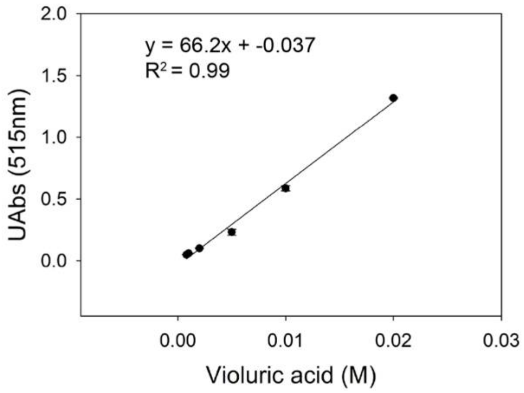

Figure S1. Determination of molar extinction coefficient of violuric acid. The assay was performed in a plate reader at 25 °C in 100 mM sodium tartrate buffer pH 4. Once substrate concentrations were fully-oxidized with HRPL PK2 [20], A515 nm were determined and fitted to a linear regression equation; ε= 113 M^{-1} cm^{-1} obtained from Beer-Lambert equation (c= 0.6 cm).

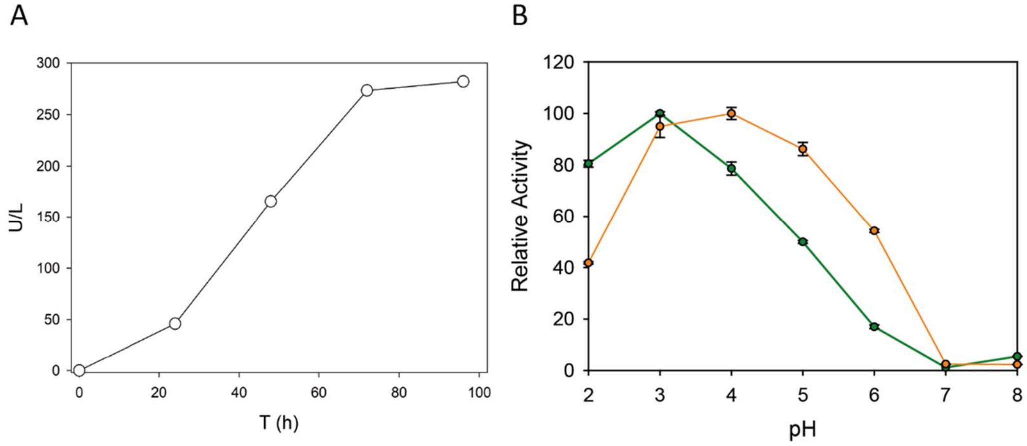

Figure S2. **A**) Time course analysis of laccase activity secreted by flask cultures of *S. cerevisiae* cells transformed with *A. pediades* laccase (ApL) fused to the mutated α_{9H2} leader, obtained in a previous laccase evolution campaign [20], as signal peptide. Laccase activity was monitored with ABTS pH 3; **B**) pH activity profiles of crude ApL with ABTS (green) and DMP (orange). Error bars correspond to the standard deviation of three replicates of each laccase variant.

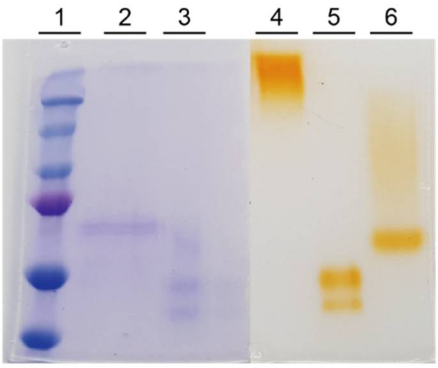

Figure S3. SDS-PAGE stained with Coomassie blue (lanes 1-3) and zymogram gel stained with DMP pH 5 (lanes 4-6) under native conditions (sample without heat-shock or β-mercaptoethanol) of native ApL (same crude enzyme than Fig. S2), compared with OB1 laccase (from PM1 basidiomycete) [13] and 3PO laccase (from *P. cinnabarinus*) [32] engineered in *S. cerevisiae.* Lanes: 1. pre-stained protein Ladder (Biorad); 2. purified 3PO laccase; 3. purified OB1 laccase; 4. crude ApL; 5. purified OB1 laccase; 6. purified 3PO laccase.

A

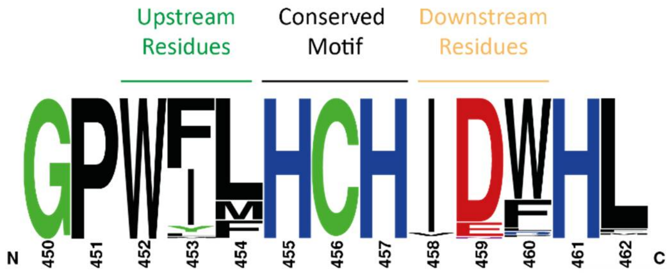

B

Position	Most frequent aminoacids	Degenerate codon
453	Phe, Ile, Leu Tyr, Met, Ala	NHS
454	Leu, Met, Phe	HTS
459	Asp, Glu, Phe Asn	DWW
460	Trp, Phe, Arg	WKS

Figure S4. Combinatorial saturation mutagenesis of 453, 454, and 459, 460 residues in DM variant. **A)** Sequence logo of the amino acid residues adjacent to the conserved HCH tripeptide obtained from the alignment of 482 Agaricales laccase sequences (numbering refers to ApL); **B)** Predominant amino acids in the positions selected for CSM and the degenerate codons used in the mutagenic primers to cover those combinations (S= CG, H= ACT, N= ACGT, D= AGT, W= AT, K= GT).

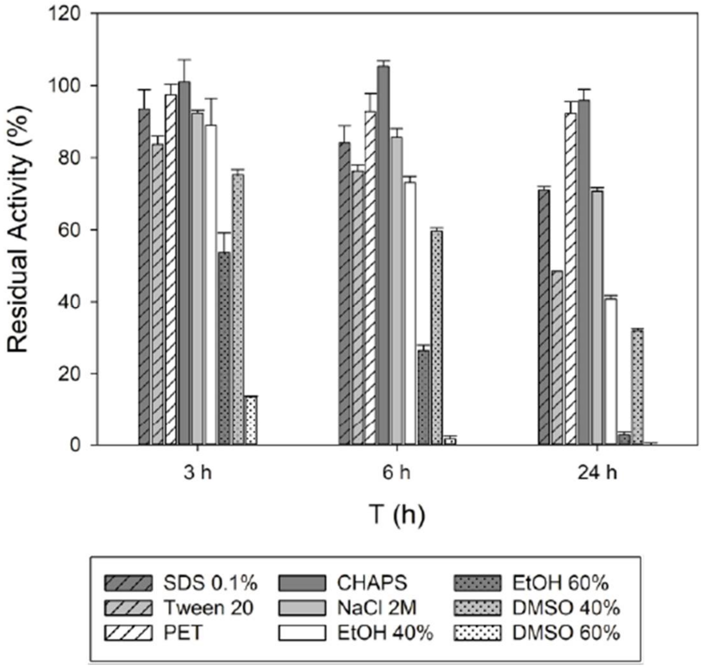

Figure S5. Stability of purified DM variant to the presence of different substances 0.1% SDS, 34 mM Tween 20, PET or CHAPS, 2M NaCl, and 40% and 60% EtOH or DMSO at neutral conditions (20 mM Tris-HCl pH 7). The residual activity was periodically measured with ABTS pH 3.

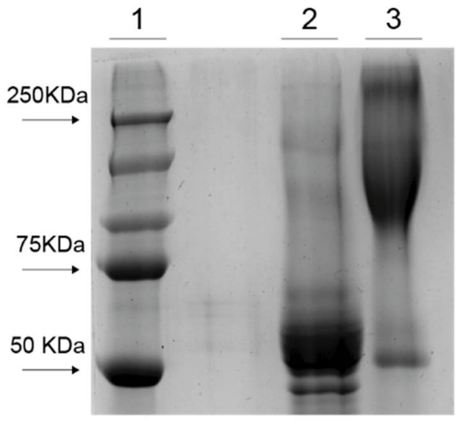

Figure S6. SDS-PAGE showing the hyperglycosylation of 7F12 laccase variant. The theorical MW of 7F12 laccase is 55 KDa. 1. Protein Ladder. 2. Purified 7F12 after deglycosylation with Endo H. 3. Purified 7F12 laccase before deglycosylation.

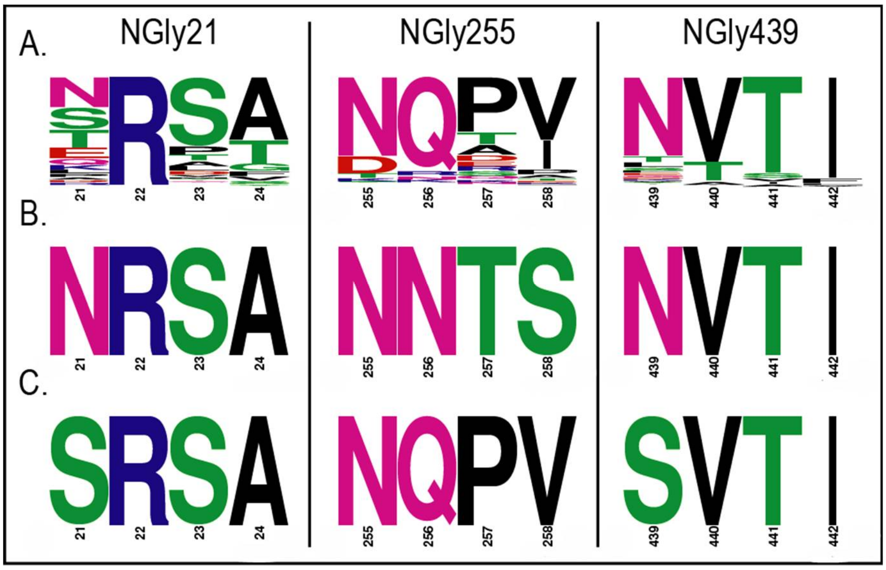

Figure S7. Sequence logos for the three putative N-glycosylation sites (N21, N255 and N439). **A)** Representation of the amino acid conservation for these sites in the multiple alignment of 482 Agaricales laccases; **B)** Amino acid sequence of ApL; **C)** Edited sequences in NGly21, NGly255 and NGly439 variants for the removal of the corresponding N-glycosylation sites in ApL by substitution with consensus amino acids.

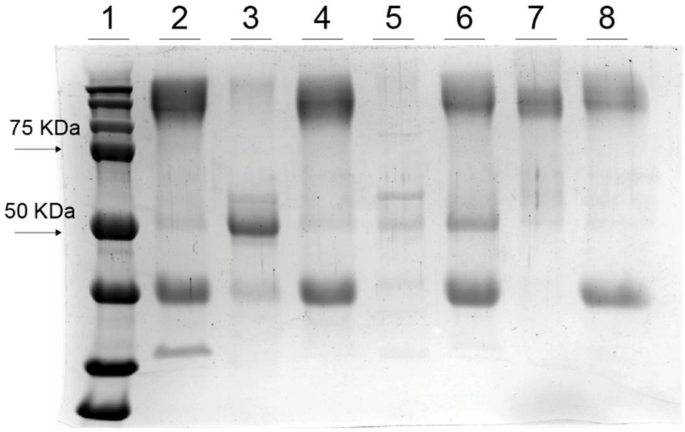

Figure S8. SDS-PAGE of 7F12 laccase and NGly variants after HiTrap Con A 4B chromatography. Lanes: 1) Protein Ladder. 2) retained 7F12 fraction. 3) non-retained NGLy21 fraction. 4) retained NGly21 fraction. 5) non-retained NGLy255 fraction. 6) retained NGly255 fraction. 7) non-retained NGLy439 fraction. 8) retained NGly439 fraction; 30 μg of protein per well.

Table S1. Sequences of the primers used.

ExtFw	5′ CTGGGGTAATTAATCAGCGAAGCGATG 3′
ExtRv	5′ GAGCGTCCCAAAACCTTCTCAAGCAAG 3′
ApL N398D Fw	5′ CAGTGGGTGACCCTCACCC 3′
ApL N398D Rv	5′ GGGTGAGGGTCACCCACTG 3′
ApL CSM (453;454) Fw	5′ ACTGATAATTCTGGTCCTTGGNHSHTSCACTGTCAT 3′
ApL CSM (453;454) Rv	5′ ATGACAGTGSADSDNCCAAGGACCAGAATTATCAGT 3′
ApL CSM (459;460) Fw	5′ ATGCACTGTCATATADWWWKSCACTTAGAAGCA 3′
ApL CSM (459;460) Rv	5′ TGCTTCTAAGTGSMWWWHTATATGACAGTGCAT 3′
Laxial ApL Fw	5′ TTAGAAGCAGGANNKGCCGTCGTATTC 3′
Laxial ApL Rv	5′ GAATACGACGGCMNNTCCTGCTTCTAA 3′
ApL NGly21 Fw	5′ CTGATGGCTTTAGTAGGTCCGCTG 3′
ApL NGly21 Rv	5′ CAGCGGACCTACTAAAGCCATCAG 3′
ApL NGly255 Fw	5′ GTTGACTACAAACCAACCAGTTGGAGATGGTAATTTCTGG 3′
ApL NGly255 Rv	5′ CCAGAAATTACCATCTCCAACTGGTTGGTTTGTAGTCAAC 3′
ApL NGly439 Fw	5′ ACGGGCCCACCAGGATCTTCTGTTACCAT 3′
ApL NGly439 Rv	5′ ATGGTAACAGAAGATCCTGGTGGGCCCGT 3′

Table S2. Thermal stabilities as T50 (10 min) assay of the retained and no-retained (nr) fractions of 7F12 laccase and its NGly variants obtained from the Concanavalin A affinity chromatography.

Laccase - glycosylation fraction	T50 (°C)
7F12	59 ± 0.5
Gly21	57.4 ± 0.4
Gly21-nr	58.7 ± 0.4
Gly255	58.4 ± 0.3
Gly255-nr	59.6 ± 0.4
Gly439	58.6 ± 0.3
Gly439-nr	60 ± 0.4
